# Post-Acute COVID-19 Rehabilitation Network Proposal: From Intensive to Extensive and Home-Based IT Supported Services

**DOI:** 10.3390/ijerph17249335

**Published:** 2020-12-14

**Authors:** Monica Pinto, Francesca Gimigliano, Stefania De Simone, Massimo Costa, Attilio A. M. Bianchi, Giovanni Iolascon

**Affiliations:** 1 Rehabilitation Medicine Unit, Strategic Health Services Department, Istituto Nazionale Tumori-IRCCS-Fondazione G. Pascale, 80131 Napoli, Italy; 2 Department of Mental and Physical Health and Preventive Medicine, University of Campania “Luigi Vanvitelli”, 81100 Caserta, Italy; francesca.gimigliano@unicampania.it; 3 Institute for Research on Innovation and Services for Development (IRISS), National Research Council of Italy, via San Felice, 80134 Napoli, Italy; s.desimone@iriss.cnr.it; 4 Physical and Rehabilitation Unit, AORN Vincenzo Cardarelli, 80131 Napoli, Italy; massimo.costa@aocardarelli.it; 5 General Manager, Istituto Nazionale Tumori-IRCCS-Fondazione G. Pascale, 80131 Napoli, Italy; a.bianchi@istitutotumori.na.it; 6 Multidisciplinary Department of Medical, Surgical and Dental Specialties, University of Campania “Luigi Vanvitelli”, 81100 Caserta, Italy; giovanni.iolascon@unicampania.it

**Keywords:** post COVID-19 care, post-acute rehabilitation, clinical governance, network model, IT

## Abstract

Management of COVID-19 post-acute syndrome is an emerging health issue in rehabilitation. This article aims to present a proposal, based on the principles of clinical governance, health management and information technology (IT), and to respond to the need for a structured organization model for post-acute COVID-19 rehabilitation. The authors present a regional-based model of a network of clinicians and healthcare managers using a dedicated IT platform to achieve both effectiveness and efficiency objectives, to ensure coordination of the available resources and the most appropriate rehabilitative treatment for patients. The proposed post-acute COVID-19 rehabilitation network has been designed according to the model of a clinical management project within the Italian national healthcare system, and its context is an easily adjustable model for the European healthcare systems. The authors base the project on current laws and scientific guidelines in rehabilitation in Italy and in Europe and use the SWOT analysis technique to assess the proposal feasibility. The primary aims of the project are: (1) standardizing the minimum assessment tools of post-COVID-19 patients with disabilities; (2) ensuring an individual rehabilitation project for each patient with international classification of functioning, disability and health (ICF) coding and (3) reporting the activity performance with appropriate indicators. The secondary aims are: (1) developing educational programs for patients and care givers also aimed at acquiring better empowerment and positive behavior; (2) creating a regional database for data collection and (3) improving IT, and specifically tele-rehabilitation, as a suitable approach during the COVID-19 emergency and also in the future. Expected results are: continuum of care; effectiveness, efficacy and appropriateness in the delivery of rehabilitation treatments through a standardized minimum assessment and the wording of the individual rehabilitation project and a precise reporting of performance indicators to measure the effectiveness of clinical activities and the satisfaction of patients and caregivers. The assessment of results will be analyzed at three and six months to implement corrective actions according to the concept of continuous improvement of the Deming cycle. The IT remote approach allows the patient to meet the needs of proximity of care and empowerment, and, at the same time, to contain the spread of infection. This project could have a significant healthcare impact ensuring a more efficient and effective management of the demand of rehabilitation post-acute COVID-19, expanding the professional skills of the rehabilitation team members, improving both clinical and process data, in addition to optimal allocation of available economic resources.

## 1. Introduction

The ongoing COVID-19 pandemic is due to a new infectious disease that causes a severe acute corona virus 2 respiratory syndrome (SARS-CoV-2). The outbreak was in China at the end of 2019, but the virus spread rapidly around the world [1] and is still dangerously active. The first cases were confirmed in Italy on 31 January 2020, in Rome, and the infection reached all the Italian regions but with a different incidence and prevalence between the north and the south of the country. According to the data from the Italian Civil Protection Department website—Presidency of the Council of Ministers, on 20 September 2020 in Italy there were 298,156 total cases, of which 35,707 died, 218,351 recovered and 44,098 were currently positive [2].

The treatment of COVID-19 post-acute patients is an emerging challenge in rehabilitation, and a new organizational design for delivering rehabilitation care to an increasing number of patients is required. This article presents the design of an information technology (IT) based network as the structured organization model for post-acute COVID-19 rehabilitation using principles and methodologies of clinical governance, health management and IT tools.

Apart from asymptomatic cases, affected patients present clinical scenarios that are different in severity and prognosis, varying from mild symptomatic cases to severe respiratory dysfunction [3] frequently worsened by clotting and cardiovascular issues [4], central and peripheral neurological complications [5] and metabolic alterations which need a prolonged hospital and intensive care unit (ICU) stay. After the resolution of the acute phase, physical, emotional and psychological impairments often persist even for a prolonged period and contribute to a complex and multi-factorial disability by requiring continuity of care and a rehabilitative multimodal management [6,7,8].

In 2017, the World Health Organization (WHO) “Rehabilitation 2030-a call for action” strengthened the central role of rehabilitation from both the public health and the clinical perspective [9]. As an essential component of the post-acute care, the rehabilitation aims to reduce long-term disability and enables patients to live in the community and to return to their previous level of social participation. The specialist in physical and rehabilitation medicine has a crucial role in coordinating the rehabilitation team and in defining the individual rehabilitation project (IRP) for each patient in accordance with the directives of the European scientific societies [10], and the Italian rehabilitation national plan [11]. The IRP includes the planned rehabilitative programs and all the health professionals involved the intensity of treatment and the setting from in-patient to out-patient. To formulate the IRP, it is necessary to carry out a multidimensional assessment, typical of the rehabilitative approach, based on measurement tools and the international classification of functioning, disability and health (ICF), an international comprehensive code system promoted by the WHO since 2001 [12,13].

The pandemic has rapidly increased rehabilitation needs, putting both the existing organization of intensive and extensive rehabilitation services in crisis. Based on a clinical governance methodology [14,15], it is strategic to have a well-designed post-acute COVID-19 rehabilitation organization to respond to the need of care and to guarantee efficacy, efficiency and appropriateness of rehabilitative treatment of post-COVID-19 patients [16,17]. The network model is an optimal strategy for the management of many complex health issues such as stroke, cancer and Lymphedema [18]. The authors present a regional-scale IT network of clinicians, rehabilitation professionals and healthcare managers designed for post-acute COVID-19 rehabilitation to achieve both effectiveness and efficiency objectives, improving the use of available resources and the delivery of the most appropriate rehabilitative treatment to each patient.

**Figure 1:**
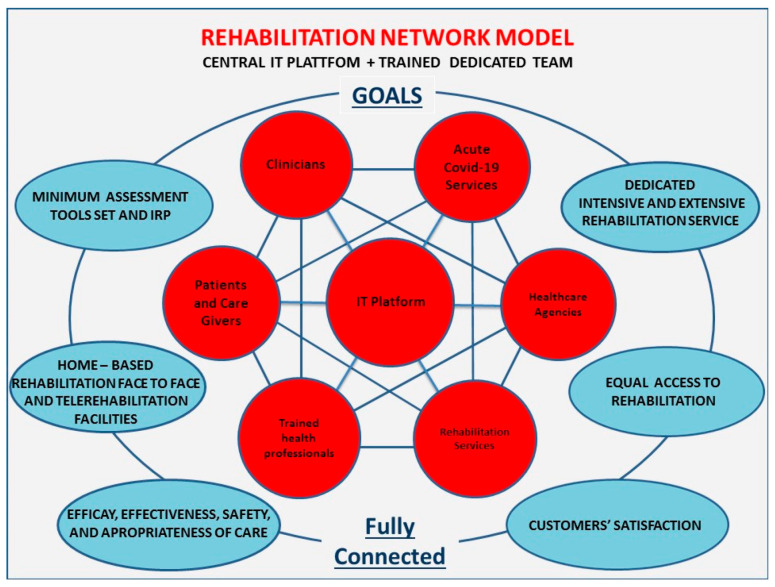
Rehabilitation Network Model.

In the COVID-19 pandemic scenario, an IT system as a network based on a dedicated IT platform would be a very useful strategy to meet the needs of very different bodies such as healthcare agencies, healthcare professionals and patients with their families. The IT strategy also includes remote assistance called telemedicine, and specifically Tele-rehabilitation, to treat selected patients able to use this approach, both for clinical conditions and personal factors, and represents an opportunity for patients and professionals to implement care services, avoid difficulties of access and reduce the infection spreading [19,20,21]. Furthermore, the IT system could provide better monitoring and reporting of the interventions through captured data flows and the optimal allocation of available economic resources. The proposed model of the IT-based network, the bodies involved, and the expected results are briefly illustrated in Figure 1.

## 2. Methods

The authors developed the proposal for a regional-scale model of a post-acute COVID-19 rehabilitation network according to clinical governance and health management principles [22,23] through a four-step process: (1) searching for current laws and scientific guidelines in rehabilitation in Italy and in Europe; (2) carrying out a feasibility study of the project using the SWOT analysis technique to identify and evaluate the factors that could affect the success of the proposal [24]; (3) drafting of the project with primary and secondary objectives, activities, indicators, timing and estimated costs, and providing for the realization of the IT platform; (4) foreseeing the main expected results and providing for continuous improvement actions according to the Deming cycle concept [25]. All procedures described are in accordance with the ethical standards of the declaration of Helsinki and the local ethics committee’s guidelines (Italian Law: DM 18/03/1998 “Linee Guida di riferimento per l’istituzione ed il funzionamento dei Comitati Etici).

Steps 1 and 2 cover the activities leading to the preliminary findings for the project drafting.

### 2.1. Step 1: Evaluation of the Current Laws and Scientific Guidelines in Rehabilitation in Italy and in Europe

For the first step, we performed a search on national and international guidelines databases and a search on the Italian ministry website looking for existing legislation on rehabilitation.

The Italian health system [26] consists of 20 regional health systems and two autonomous provinces health systems that define the organization of care independently from each other. In 1998, the Italian guidelines for rehabilitation activities [27] provided for three levels of post-acute settings which are still in use, based on the number of hours of rehabilitation and on the needs of medical and nursing care:

- High intensity in-patients (rehabilitation 3 h/day, 24 h medical and nursing care);
- Extensive intensity in-patients (rehabilitation 1 h/day, 24 h medical and nursing care);
- Ambulatory or home-based outpatients (rehabilitation 30–60 min/day, no 24 h care).

In addition, these categories include other specific settings such as the day hospital (that provides rehabilitation 3 h/day but limited hours of medical and nursing care, considered as intensive in-patient care), and residential and semi-residential rehabilitation (considered extensive in-patient care). These last settings, although important, have not been considered in the starting phase of the project but they will be included in a successive phase. Most recently the 2011 rehabilitation national plan stated that the rehabilitation activities are based on the biopsychosocial model of the ICF and on the wording of the individual rehabilitation plan (IRP) which includes the objectives, timing and interventions to meet the physical, mental and social needs of patients.

According to the 2011 Italian rehabilitation national plan [11] the authors consider the ICF the most appropriate coding system in rehabilitation in general [28,29] and in post-acute COVID-19 rehabilitation too. Post-acute COVID-19 rehabilitation provides a comprehensive approach to all aspects of health, and the ICF model can capture the complexity of biological, individual and social aspects of human health, and could also categorize clinical outcomes and outcome measures [30]. In the COVID-19 pandemic the Italian rehabilitation services have changed their organization and working methods [31]. Some rehabilitation departments were converted to COVID-19 acute wards due to the need of beds for symptomatic cases. Only now are they opening again, frequently with a reduced availability of beds and outpatients’ reservations. Rehabilitation facilities have taken steps to continue assistance for people with disabilities and to prevent contagion from COVID-19 at the same time, in opportune cases with IT support [32,33]. However, there is no legislation and strong guidelines concerning the rehabilitation network at the time of discharge from COVID-19 acute wards.

### 2.2. Step 2: SWOT Analysis

**Figure 2:**
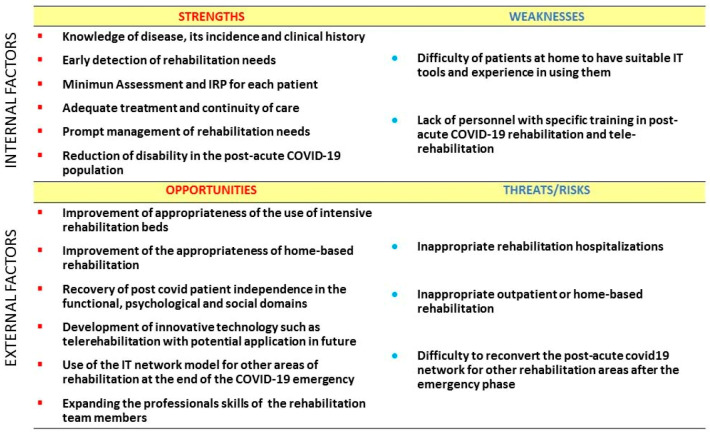
SWOT analysis of the post-acute COVID-19 rehabilitation network model.

After investigating the existing regulatory aspects, we carried out a feasibility study using SWOT analysis to identify and evaluate the factors that could affect the success of our proposal. SWOT is an acronym that stands for strengths, weaknesses, opportunities and threats and these categories are usually represented in a two by two matrix. SWOT analysis is a strategic planning technique taken from the business world and used by individuals or collaboration groups to analyze the factors that can strategically influence the implementation of a new project in the preliminary phase of decision process. It is used to uncover the optimal match between the internal factors, strengths and weaknesses, and the external factors, opportunities and threats, of a project. SWOT analysis can be usefully applied in medicine and health systems to evaluate the favorable and unfavorable aspects of a project [34]. According to the SWOT analysis methodology the authors brainstormed the main influencing factors of the proposal among the categories of strengths, weaknesses, opportunities, threats/risks. Figure 2 shows these factors by two matrix of the SWOT analysis carried out for the network model.

### 2.3. Step 3: Drafting the Project

Based on the results of steps 1 and 2, the third step consists of drafting the project, with specific primary and secondary objectives, activities, indicators, timing and estimated costs, and providing for the realization of an IT platform where the different bodies (clinicians, health professionals, health managers, patients and caregivers) can be connected in real time, realizing the concept of the network. Networking is becoming a winning model in many fields including health care. It allows all parties to interact more quickly and to better allocate human and economic resources. There are already healthcare dedicated networks such as the oncology network in Campania region and emergency health care networks spread in many countries with structured health systems. The network model facilitates quick access to the available resources such as hospital beds or outpatient treatments and, therefore, the continuity of care from hospital (acute care) to rehabilitation (post-acute care hospitals, territorial services, and patient residence services), ensuring the correct clinical data transmission and reporting activities.

### 2.4. Step 4: Expected Results and Improvement Actions

This article presents a proposal for an IT-based network as the regional-level organization model for post-acute COVID-19 rehabilitation. Therefore, the fourth step consists of foreseeing the main expected results and providing for continuous improvement actions.

**Figure 3:**
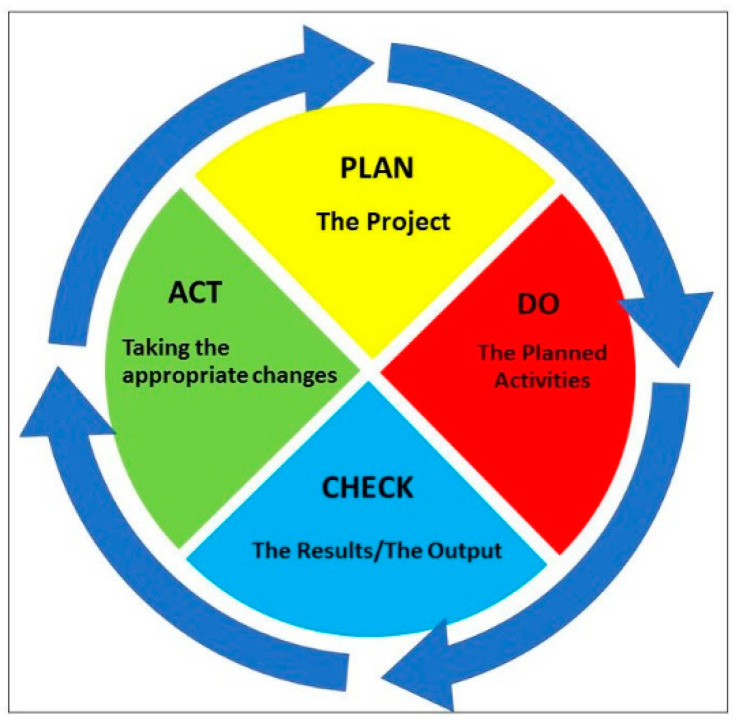
Deming Cycle.

This proposal is a living document, so continuous quality improvement is crucial to achieve success. The Deming cycle is a quality improvement model, well assessed both in research and business fields, that can be usefully applied in many contexts including healthcare management and rehabilitation [35]. It consists of four steps: plan the project; do the planned actions; check the results or output and act, taking the appropriate change resolutions (Figure 3).

The Deming Cycle, also called plan, do, check, act (PDCA) cycle as an acronym of its four steps, is specifically suitable for our project because of the changing pandemic scenario in which it is located. Steps three and four methodology are described in the present Methods section, but the findings are described in detail in the Results section.

## 3. Results

### 3.1. The Project Plan

Based on the findings of step one and on the SWOT analysis of step two, the authors formulated the post-acute COVID-19 rehabilitation network proposal. The implementation of a regional IT platform with specific applications, and managed by a trained dedicated team, is essential to guarantee the success of the network. However, this is a very technical aspect of the project and the authors have preferred not to address it in this article because it is oriented to the aspects of clinical governance and healthcare management of the proposal rather than the technological components.

The network enables clinicians, rehabilitation professionals, patients and services to connect in real time using IT platforms. It would guarantee:

- Wide coverage of the post-acute COVID-19 target population in the region;
- The homogeneous evaluation of patients by validated assessment tools;
- The promptness of access to care and the availability of tailored care settings according to the complexity of the demand;
- Limiting the movements of people, also adopting in clinical practice IT applications such as remote access.

The multidisciplinary post-acute COVID-19 rehabilitation team should include all professional figures necessary, each one for their competence, including an IT specialist. The provision of rehabilitation aids to patients should include electronic equipment and remote monitoring devices if necessary, as well as other aids and orthotics. These aids will be returned at the end of treatment, subjected to sanitization according to current rules, and then reused. Personal protective equipment (PPE) for both health professionals and patients has been adopted according to the current national and regional laws and recommendations.

Specific rehabilitation programs are necessary to manage the post-acute COVID-19 limitations in the following functional and activities domains: (1) respiratory functions, with particular attention to cardio-respiratory endurance and respiratory pattern; (2) neurocognitive functions with particular attention to memory deficits, sensory-motor central impairments, central and peripheral neuropathies and dysphagia; (3) motor functions including muscle strength and endurance, flexibility, joint active motion and stability also considering the preexisting clinical and general functional status; (4) emotional and psychological functions linked to the traumatic experience of the disease.

### 3.2. The Objectives

In order to guarantee efficiency, efficacy, safety and appropriateness in the rehabilitation management of the patient suffering from disability due to COVID-19, the present project proposal identifies the primary objectives as follows:

(1) Standardizing the minimum assessment tools set (Table 1) both for people being discharged from hospital and for those at home with the use of international validated tools (Table 1);
(2) Planning the IRP for each patient including the rehabilitation treatment setting (intensive, extensive, both ambulatory or home-based, in person or remote), timed programs, objectives identified by the appropriate ICF codes, and the team of professionals involved to guarantee efficacy, efficiency and appropriateness of care and patient satisfaction;
(3) Reporting the performance of the activities by indicators (Table 2) at three and six months:
  (a) Process indicators: waiting time in days for the first assessment, number (No.) of assessments based on the minimum tools set/No. of total assessments, No. of individual rehabilitative plans/No. of patients treated in all settings;
  (b) Outcomes indicators: changes in Modified Barthel Index scores, Health Survey SF 12 scores (patients versus general population), EuroQual EQ 5D scores (patients versus general population), No. of adverse events during Tele-rehabilitation/No. of adverse events during home-based rehabilitation face to face plus remote.

One of the primary objectives of the project is to develop standardized minimum assessment tools set for patients cured from COVID-19 and with disabilities, to be applied as early as possible during the acute hospital stay or, at the latest, before discharge from an acute setting. It is really important to have precise criteria for the safe discharge of post acute COVID-19 patients from the acute wards towards rehabilitation [32]. The consensus paper by Jin X. et al. [36], produced a core outcome set for clinical trials on patients with COVID-19, the COS-COVID; the core set consists of different outcomes, but only one is specific for rehabilitation and namely for pulmonary function. Therefore, the project aims to identify the minimum assessment tools set that covers the main disabilities areas for these patients.

Planning the IRP for each patient is another primary objective. It uses the parameters of impairment, activity limitation and restriction of social participation as listed in ICF. The peculiarity and novelty of the corona virus pathology requires specific attention in considering the significant ICF categories for each patient to plan the rehabilitative interventions. For example, consideration of impairments such as respiratory function and swallowing are mandatory for the correct management in a rehabilitation setting. The minimum assessment tools set is based on the current recommendations present in the most recent literature [37,38] and all the tools are validated (references are reported in Table 1). In the absence of evidence, the selection criteria are based on general clinical practice. The same consideration can be made for the choice of rehabilitation programs.

The indicators of clinical performance, both process indicators and outcomes indicators, are the third main objective, and they are essential for monitoring the project activities and allowing adjustments in time to get optimal outcomes.

Secondary objectives of the project are:

- Educational programs for patient and caregiver also aimed at acquiring better empowerment and positive behaviors such as stopping smoking (pre-habilitation);
- Creation of a regional database for data collection;
- Improving IT, and specifically Tele-rehabilitation, by training the rehabilitation staff in the use of telemedicine devices, implementing facilities and clarifying legal aspects.

In critical times like the COVID-19 pandemic, IT can support, without ever replacing, human resources in medicine, and there are already experiences of Tele-rehabilitation in different fields [39]. In our proposal, a specific consent to Tele-rehabilitation should be provided and signed by the patient and caregiver, including the acceptance of the availability of the caregiver at home during the treatment sessions, the use of the institutional network platform, the risks regarding personal data protection, and the impossibility of managing side effects without emergency. Home patients will be provided with a pulse-oximeter, pedometer, and IT aids or an app on a smart-phone for remote evaluation of daily physical activity. Furthermore, written indications in case of emergency will be provided, for example: “in case of dyspnea with SpO2 < 92, please stop the exercise you are doing and rest 5–10 min, and if the dyspnea does not stop, please alert your doctor or call Emergency”; and “in case of pain during the exercise, please stop the exercise and rest, and if the pain does not resolve itself in 5–10 min, please alert your doctor or call Emergency”. Tele-rehabilitation is not an alternative to treatment in person but can be complementary. In the case of difficulties for the patient in following the programs remotely, or in case of unsatisfactory results, the treatment will be promptly converted into the mode in person.

### 3.3. Expected Results and Improvement Actions

The assessment of results will be carried out at three months and six months, and corrective actions will be planned through the Deming Cycle model.

Based on the network proposal, we can expect to guarantee to post-acute COVID-19 patients with disabilities, consequent to the disease or its treatments, the best possible recovery of individual skills and social participation through a multidisciplinary, multi-professional and continuity care path with significant technological, economic and social impact. Specifically, we can expect:

- Dedicated post-acute COVID-19 intensive and extensive rehabilitation beds, which in every case can be totally or partially reconverted to general rehabilitation at the end of the COVID-19 pandemic;
- Easy access to rehabilitation care;
- Timely rehabilitation management also for patients at home;
- Continuum of care and appropriate levels of care in the different settings;
- Improvement of the quality perceived by patients (customers satisfaction) with the empowerment of patients and their family;
- Monitoring the activities by performance indicators (Table 2) and managing challenges by the Deming plan-do-check-act cycle to improve clinical and organizational appropriateness and quality of care;
- Compliance with COVID-19 guidelines to prevent and contain the infection spread (social distancing, and appropriate use of PPE);
- Creation of regional database for collecting data to support future planning decisions in the field.

We hope to meet these optimal results in six months with a three months first milestone (see Table 2).

In particular we expect that hospitalized patients with multi-organ involvement and severe disease will mostly require intensive or extensive rehabilitation, and home-treated patients will be able to continue rehabilitation in the home setting with only a few exceptions requiring an intensive or extensive setting. Therefore, depending on the different rates of home patients, hospitalized patients including ICU patients, and on the number of patients healed, each region can plan the number of intensive and extensive rehabilitation beds in order to meet the expected demand. However, for most patients, the appropriate intervention strategy will be home rehabilitation that can be managed in the two modalities, in person or by Tele-rehabilitation, not necessarily alternative but also concurrent or subsequent.

Furthermore, in order to limit the risk of the spread of the contagion, we exclude the day-hospital and the ambulatory settings in the starting phase as well as the residential and semi-residential setting, but we consider they may be included in the network offers in the future, according to the Deming Cycle for continuous quality improvement. Our approach results in the optimization of the risk-benefit ratio (including the contagion risk for both patients and health professionals) with a significant reduction in direct and indirect costs.

## 4. Discussion

The innovative aspects of the project are both the network organization and the possibility to manage the home-based rehabilitation in two ways: as usual with the therapist in person at the patient’s home, or by remote with the therapist supervised IT-assisted way, also called Tele-rehabilitation. As already mentioned in Methods, the network model is a very well assessed organizational model in many fields of medicine and healthcare management, such as emergency, cancer care and also rehabilitation, while Tele-rehabilitation has not been sufficiently promoted.

Tele-rehabilitation, even if it cannot completely replace conventional rehabilitation, has a potential application particularly suitable for the current emergency situation. Several studies have evaluated the possibility of using technology (telephone, internet etc.) for an alternative or additional connection method between patients and healthcare professionals [39,40,41,42], and the experience in using this method for other pathologies could be useful for people cured from COVID-19. The authors have drawn important suggestions from the experiences of New Zealand and South Korea [43,44,45]. Although these countries are very different from Italy in geographical and cultural features, their frontier technology and optimal healthcare systems and rehabilitation organization during the COVID-19 pandemic supported the drafting of this project.

The COVID-19 emergency quickly imposed a huge change in the health system organization and has brought about new crises. As always it can be seen that the standardization of care improves the effectiveness, the efficiency and the appropriateness of services. A rehabilitation network for post-acute COVID-19 patients will guarantee equality of access and evaluation to all patients, uniformity of care, taking into account any personal peculiarities and family environment. The first step towards getting these optimal results is to define the minimum criteria for the safe discharge of post-acute COVID-19 patients from hospital to a specialist facility or home. Moreover, it would allow better monitoring of interventions and reporting through shared data flows. In our opinion, it is crucial to adopt specific actions aimed at organizing the rehabilitation network for post-acute COVID-19 patients; in this way we can guarantee the effective management of rehabilitation demand, customer satisfaction and the proper allocation of available economic resources. Specifically, Tele-rehabilitation may play a central role in the change in delivering rehabilitation activities and in the inclusion of patients with restricted access to rehabilitation, such as post-acute COVID-19 patients. Of course, older people may have difficulty in using IT devices. However, the Italian Higher Institute of Health [46] has recently indicated the proportion (%) of COVID-19 cases by age groups: 0–18 years 15%; 19–50 years 46.8%; 51–70 years 26.1%; >70 years 12%. Mortality is higher in people > 50 years. Therefore, the proportion of COVID-19 survivors under 70 years can justify an investment in IT rehabilitation. 

We estimated that a regional network IT platform has an initial cost of about 300,000 Euros, the annual cost of the software update is approximately 10,000 Euros and the annual cost of a small regional supportive team of four people is about 100,000 Euros. Each local health authority has to sustain the costs of updating their systems. Although there are still barriers, such as the availability of technological instruments at the patients’ home and a possible lack of IT competence, we already have the model of the oncology network in Campania Region that provides extremely simple access with a very short training in the context of all healthcare professionals (nurses, physiotherapists, doctors) ordinary daily work and patients’ daily activities by using computers, tablets and/or smart-phones. Furthermore, in the future, Tele-rehabilitation will be able to expand the offer of assistance to patients who experience barriers to access care facilities. Of course, many aspects of Tele-rehabilitation are still under observation, especially personal data protection and legal implications if any side effects or complications should occur. To limit adverse events and any legal disputes it is important to give written information to be followed in case of adverse events (such as pain) and to obtain an informed written and detailed consent from the patient. Another criticism of our proposal is represented by not having involved the project stakeholders, represented by patients and their relatives, in the development of the proposal, as is usual. The stakeholders should certainly be involved in the process of revision in the near future. Finally, although the IT platform is a central element of the project, it is also a very technical aspect of the project, and the authors have preferred not to address it in this article. However, they reserve the opportunity to present the platform structure in a dedicated article.

## 5. Conclusions

This project aims to support healthcare systems, specifically the Italian regionally-based healthcare system, and the patients cured from the COVID-19 infection, by offering an organization model of a dedicated network for post-acute COVID-19 rehabilitation which enables:

- Continuity of care from an acute hospital stay to post-acute inpatient and outpatient or home-based rehabilitation care, patients’ assessment based on a minimum assessment tools set and early onset of the treatment of disabilities;
- Optimal clinical governance, even in emergency conditions, in terms of equal access to care, efficiency, efficacy, safety and appropriateness of rehabilitation treatments;
- Technological promotion in IT health care, and specifically in Tele-rehabilitation.
- Customers’ satisfaction in terms of reduction of disability and improvement of quality of life.

This model offers the advantage of being applicable in different contexts, not only in the Italian regions but also in other countries and highlights the importance of communication and of the working team including clinicians, rehabilitation professionals, healthcare managers, patients and caregivers in the pursuit of the objectives of clinical governance and healthcare management. Rehabilitation is strategic in the context of healthcare strategies oriented to the burden management of post-acute and chronic diseases. The ongoing COVID-19 infection, although it is an acute disease, is assuming some characteristic of sub-acute diseases including persistent disabling outcomes and growing needs of rehabilitative treatments. The innovation of the project consists in adopting an emergency management strategy based on a web connection to manage the rehabilitative needs in real time in the COVID-19 pandemic scenario. IT can also support the home care setting by Tele-rehabilitation, considered not alternative, but complementary, to the treatment in person. Our proposal can, therefore, contribute to healthcare decisions and clinical governance strategies both at the regional and national level in Italy as well as in other countries.

## Figures and Tables

**Table 1:** Minimum assessment tools set.

	Tool	Scoring	ICF	Reference
IMPAIRMENTS				
Pain	Numeric Rating Scale	0–10	b280	Farrar JT et al., Pain 2000
	Douleur Neurophatique 4 questions	0–10	b280	Bouhassira D et al., Pain 2005
Anxiety	Hospital Anxiety and Depression Scale	0–21	b152	Zigmond AS et al., Acta Psychiatr Scand. 1983
Dyspnea	Modified Dyspnea Borg Scale	0–10	b460	Muza SR et al., Am Rev Respir Dis. 1990
Muscle strength	MRC muscle Testing	0–5	b730	Medical Research Council, Memorandum no.45 1976
Dysphagia	Three oz-Water Swallow Test	Yes/no	B510.5	De Pippo KL et al., Arch Neurol. 1992
Fatigue	Fatigue Severity Scale	mean score	b455	Krupp LB et al., Arch Neurol. 1989
ACTIVITIES				
Mobility and Walking	Timed Up and Go Test	≤12 ss	b510	Podsiadlo D et al., J Am Geriatr Soc. 1991
	6 Minute Walking Test	meters/6 min	b450	ATS, Am J Respir Crit Care Med. 2002
DISABILITY				
Activities of Daily Living	Modified Barthel Index	0–100	d450< x > d560	Shah S et al., J Clin Epidemiol. 1989
PROMs (Patient Reported Outcomes Measures)				
Quality of Life	SF-12	Physical (PCS) and Mental (MCS) composite scores 0–100		Gandek B et al., J Clin Epidemiol. 1998
	EQ-5D	Score 1 (no problem) to 3 (the worst problem) in the 5 items		EuroQoL Group, Health Policy 1990 Rabin R et al., www.euroqol.org 2011

**Table 2:** Primary objectives, indicators, and expected results.

Objectives	Indicators	Expected Results at 3 Months	Expected Results at 6 Months
Minimum assessment tools set	Waiting times for the 1st assessment	≤2 days	≤1 day
	No. assessments based on minimun tools set/No. total assessments	90%	95%
Individual Rehabilitative Plan (IRP)	No. of IRPs/No. of patients treated	90%	95%
Modified Barthel Index	scores 0–100	≥60	≥85
SF-12	Physical (PCS) and Mental (MCS) composite scores 0–100	≥35	≥45
EQ-5D-3L	Frequency distribution of the 5 items responses from 1 (no problem) to 3 (the worst problem)	1 ≥ 80%	1 ≥ 95%
		2 ≤ 25%	2 ≤ 4%
		3 ≤ 5%	3 ≤ 1%
No. adverse events during Telerehabilitation	No. adverse events during Telerehabilitation/No. adverse events in home-based rehabilitation face to face + remote	≤50%	≤50%
